# Green Synthesis of Silver Nanoparticles with *Hyssopus officinalis* and *Salvia officinalis* Extracts, Their Properties, and Antifungal Activity on *Fusarium* spp.

**DOI:** 10.3390/plants13121611

**Published:** 2024-06-11

**Authors:** Lina Dėnė, Simona Chrapačienė, Greta Laurinaitytė, Aira Rudinskaitė, Jonas Viškelis, Pranas Viškelis, Aistė Balčiūnaitienė

**Affiliations:** 1 Laboratory of Biochemistry and Technology, Institute of Horticulture, Lithuanian Research Centre for Agriculture and Forestry, Kaunas District, LT-54333 Babtai, Lithuania; jonas.viskelis@lammc.lt (J.V.); pranas.viskelis@lammc.lt (P.V.); aiste.balciunaitiene@lammc.lt (A.B.); 2 Laboratory of Plant Protection, Institute of Horticulture, Lithuanian Research Centre for Agriculture and Forestry, Kaunas District, LT-54333 Babtai, Lithuania; simona.chrapaciene@lammc.lt (S.C.);

**Keywords:** *Salvia officinalis*, *Hyssopus officinalis*, silver nanoparticles, green synthesis, *Fusarium*, inhibition

## Abstract

Recent focus has been given to nanoparticles as an alternative fungicidal compound instead of chemical ones. More environmentally friendly ways of synthesis are the highest priority regarding the antifungal agents in the agriculture sector. Therefore, in this research, hyssop (*H. officinalis*) and sage (*S. officinalis*) aqueous extracts were prepared and used as a reducing source in the green synthesis of silver nanoparticles (AgNPs). Aqueous extracts and green synthesized AgNPs were examined for phytochemical composition and antioxidant capacity. Hyssop and sage extracts based AgNPs were analyzed using UV-vis spectrometry, SEM-EDS, and TEM-EDS. Antifungal activity against *Fusarium* spp. isolates collected from different infected crops was determined. *Fusarium* spp. isolates from strawberry, asparagus, pea, carrot, wheat, and rapeseed samples identified at the molecular level by translation elongation factor 1-alpha (TEF1α) gene amplification and sequencing. Green synthesized AgNPs had lower phytochemical content, however higher antioxidant activity compared to pure extracts. Both hyssop and sage extracts are suitable reducing agents for AgNPs formation, and sage extract results in larger particle size. Aqueous hyssop extract had higher antifungal activity than aqueous sage extract. However, a 10% concentration of whole sage extract based AgNPs solution, added to the PDA medium, and a 5% concentration of hyssop extract based AgNPs inhibited *Fusarium* spp. the most. *F. proliferatum* was the most sensitive to all treatments among the other fungi.

## 1. Introduction

Nanoscience is widely applied in various industries and medicine fields, and its application in agricultural sciences has attained a special focus. The globalization of agricultural product trade highly influences pathogen occurrence, which threatens our agroecosystems [1]. Regarding the commitment to reducing synthetic pesticide use, nanoparticles (NPs) are examined as alternative antifungal agents against fungal pathogens [2]. Various NPs synthesized chemically were investigated against horticultural fungi *Alternaria alternata* (Fr.) Keissl., *Rhizoctonia solani* J.G. Kühn and *Botrytis cinerea* Pers. [3]. Different sizes of NPs inhibited *Fusarium oxysporum* f. sp. *radicis-lycopersici* J. & Sh. strains [4]. Several action modes were determined for antifungal activity against fungicide-sensitive and fungicide-resistant *F. graminearum* strains. These include fungal development and cell membrane disruption, perturbation of cellular energy utilization, and metabolism pathways [2]. However, chemically synthesized NPs are unattractive due to the usage of generally toxic and biologically harmful reducing agents [5]. Contrariwise, green synthesis of NPs using plant extracts is environmentally friendly, non-toxic, and lower in cost than physical or chemical approaches [6,7,8,9].

Plants can produce various secondary metabolites such as alkaloids, glycosides, terpenoids, saponins, steroids, flavonoids, tannins, quinones, and coumarins. Many of these phytochemicals have beneficial effects on human health as well, effectively treating various diseases [10]. Additionally, extracts obtained from a broad spectrum of plants were proven to have inhibitory effects against horticultural fungal pathogens in vitro [11,12,13,14]. For example, different extracts of *Hyssopus officinalis* L. and *Salvia officinalis* L. were shown to be rich in bioactive compounds, which contribute to the anti-fungal effect [15,16,17,18]. *H. officinalis* is a well-known medicinal and aromatic herb mainly used in the pharmaceutical and food industries. Isopinocamphone, pinocamphone, and β-pinene are the most characteristic constituents in *H. officinalis* extracts (HysO), expressing strong antifungal effects [15,16]. *S. officinalis* has long been used as an anti-inflammatory and antimicrobial agent, as its extracts have hypoglycemic and antimutagenic effects. The main biologically active components of this plant are essential oils that contain a lot of ketones: α-thujone and β-thujone [18]. Antimicrobial and antioxidant activity of *S. officinalis* extracts (SalO) are determined by phenolic compounds like flavonoids and terpenes [18,19,20].

Silver is considered to control various plant pathogens, and silver nanoparticles (AgNPs) have a high antimicrobial effect [21]. Green synthesis of AgNPs is a nonpath-ogenic single-step method and produces a considerable number of metabolites [22]. Plant secondary metabolites can act as both reducing and stabilizing agents [23] in the synthesis, and phytochemicals such as phenolic and flavonoid compounds may impart different biological activities to NPs [24]. Moreover, plants characterized by antifungal properties hold great potential in creating green synthesized silver NPs (AgNPs) with strong antifungal activity. AgNPs synthesized by plant extracts had effective anti-oomycete and antifungal activity against *Pythium aphanidermatum* (Edson) Fitzp., *Paecilomyces formosus* (accession no. MK638999), *F. oxysporum* f.sp. *cumini* Prasad & Patel, *Macrophomina phaseolina* (Tassi) Goid., and *B. cinerea* with significant inhibition rate of mycelial growth [25]. Previous research of plant extracts mediated AgNPs demonstrated rich biological composition and high biological activity [26,27].

Representatives of the *Fusarium* (Hypocreales, Ascomycota) genus rank as the most critical plant pathogens responsible for many economically destructive diseases, including vascular wilts, stems, crowns, and root rots [28]. Furthermore, some *Fusarium* species can produce secondary metabolites that belong to mycotoxins, which contaminate crops and are later passed to animals and finally to humans [29]. *Fusarium* fungi are commonly found as a complex in various agricultural plants, and their pathogenicity may be impacted by separate species existing in the complex [30]. Therefore, it is essential to investigate the sensitivity of *Fusarium* spp. occurring in plants, fruits, and berries, even in the symptomless form. Some evidence of antifungal activity of AgNPs against *Fusarium* spp. could be found: AgNPs obtained using *Pseudomonas poae* CO strain demonstrated strong antifungal activity against pathogenic *Fusarium graminearum* Schwabe [31]. Meanwhile, AgNPs synthesized by plant extracts significantly decreased the colony formation of *F. oxysporum* Schlecht. [32]. Also, reduced growth of various pathogenicity *F. oxysporum* f. sp. *cepae* Hansen [33] and *F. oxysporum* f.sp. *cumini* Prasad & Patel [25] was determined. Promising results found in the literature suggest the potential of AgNPs to inhibit various *Fusarium* isolates.

Green synthesis of AgNPs using plant extracts as reductors results in a liquid, convenient form for further application in plant protection. Plant extracts rich in biologically active compounds provide a promising source of antifungal agents with unique properties when used in the green synthesis of NPs. The antifungal potential of green synthesized AgNPs, mediated by HysO and SalO, has not yet been widely reported. Therefore, in this study, we aimed to perform green synthesis of AgNPs using *H. officinalis* and *S. officinalis* extracts, evaluate the properties of pure extracts and produced AgNPs, and determine the antifungal activity on *Fusarium* spp., isolated from the agricultural crops.

## 2. Results

### 2.1. Phytochemical Composition of Prepared Extracts and AgNPs

The pharmacological and antimicrobial activity of the extracts is determined by the biologically active compounds in the composition—flavonoids, phenolic acids, vitamins, trace elements, fatty acids, etc. Therefore, to evaluate the phytochemical composition, the quantitative composition of total phenolic compounds, flavonoids, proanthocyanidins, and hydroxycinnamic acid derivatives of *H. officinalis* (HysO) and *S. officinalis* (SalO) extracts and obtained AgNPs were evaluated. The results of the research are presented in Table 1.

The highest content of phenolic compounds was found in HysO (1.17 mg GAE/g), whereas a slightly lower amount was detected in the SalO. Also, HysO contains a larger number of flavonoid compounds compared to SalO; however, there is a lower amount of proanthocyanidins. Meanwhile, SalO contains a larger amount of hydroxycinnamic compound compared to HysO, around 40%.

The total content of proanthocyanidins, hydroxycinnamic acid derivatives, phenolic compounds, and flavonoids in HysO-AgNPs and SalO-AgNPs is lower compared to pure HysO and SalO extracts, but the character of changes is the same. A higher content of total proanthocyanidins and hydroxycinnamic acid was found in SalO-AgNPs. Meanwhile, slightly higher contents of phenolic compounds and flavonoids were detected in the HysO-AgNPs. The content of flavonoid compounds in the case of AgNPs is 13–40% lower than that of pure plant extracts. This decrease in biologically active compounds indicates that the green synthesis of silver nanoparticles has occurred. These biologically active compounds participate as reducing agents in the green synthesis of silver nanoparticles and act as a capping agent that prevents the formation of agglomerates. It is assumed that polyphenols’ donating potential facilitates the formation of nanoparticles by bioreducing Ag^+^ to Ag^0^ and additional stabilizing nanoparticles.

### 2.2. Antioxidant Capacity of the Extracts and AgNPs

**Figure 1:**
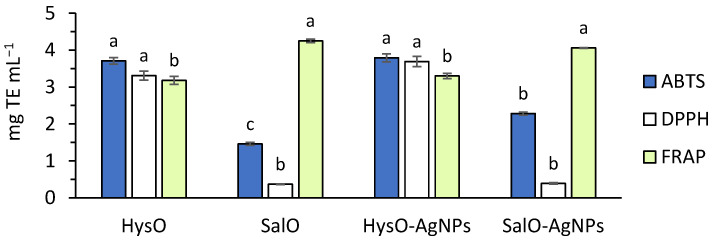
Antioxidant capacity of *S. officinalis* (SalO), *H. officinalis* (HysO) extracts, and silver nanoparticles synthesized by *S. officinalis* extract (SalO-AgNPs) and *H. officinalis* extract (HysO-AgNPs). According to Duncan’s multiple range test, different letters indicate statistical differences between the means (*p* < 0.05).

Plant extracts are mixtures of secondary metabolites whose antioxidant activity is determined by different biological mechanisms. Thus, in this study, we determined antioxidant and reducing activity of HysO and SalO extracts and the obtained green silver nanoparticles by three different methods. Results are presented in Figure 1.

Biosynthesized AgNPs antioxidant capacity was similar to the extracts used as reductors. HysO-AgNPs slightly increased activity against DPPH• compared to HysO, while SalO-AgNPs had higher activity against ABTS•+ compared to SalO. None of the investigated AgNPs had higher than pure extracts (HysO and SalO) ferric-reducing activity (FRAP).

### 2.3. Structural and Qualitative Analysis of Silver Nanoparticles

**Figure 2:**
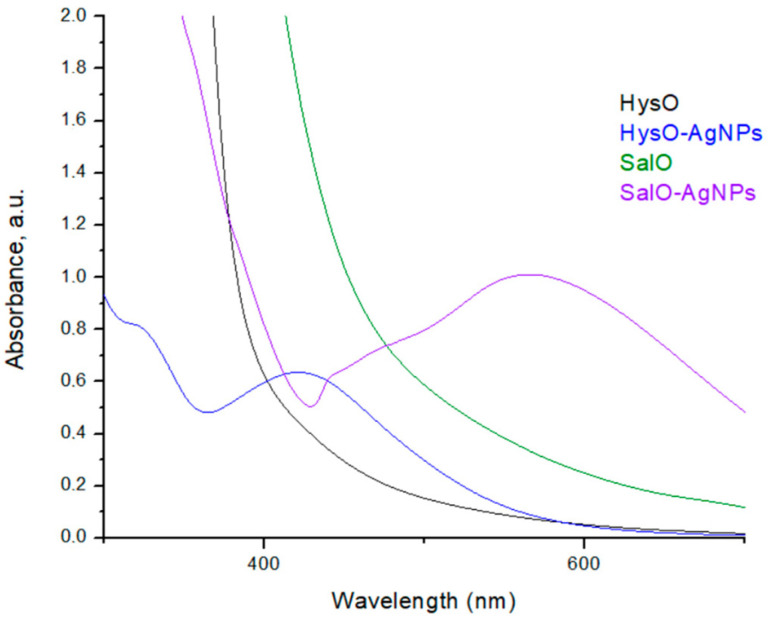
UV-vis absorption spectra of *H. officinalis* (HysO) and *S. officinalis* (SalO) extracts and silver nanoparticles synthesized by *H. officinalis* extract (HysO-AgNPs) and *S. officinalis* extract (SalO-AgNPs).

The biosynthesized silver nanoparticles that were stabilized by HysO and SalO showed a strong localized surface plasmon resonance (LSPR) peak at 565 and 423 nm (Figure 2). Typically, the intensity and position of LSPR depend on the size and shape of nanoparticles and the composition of the surrounding medium [34]. Smaller nanoparticles are proposed to absorb light primarily and have peaks near 400 nm while larger spherical particles exhibit increased scattering and have peaks at longer wavelengths [35]. Based on UV-vis absorption spectra, obtained SalO-NPs were larger than HysO-NPs.

**Figure 3:**
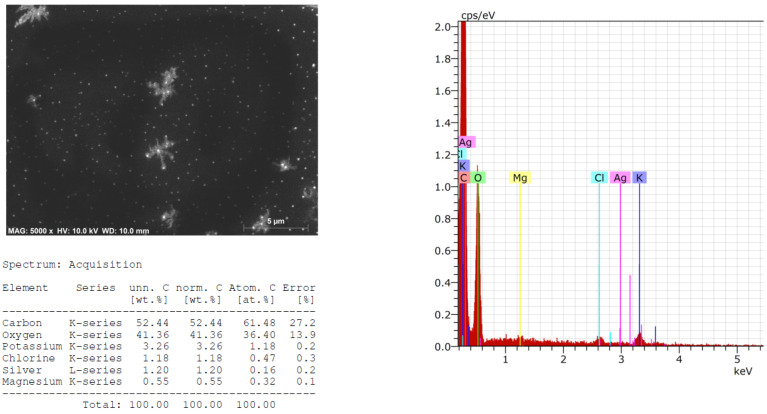
SEM images and EDS spectra of AgNPs synthesized using extract of *H. officinalis*.

**Figure 4:**
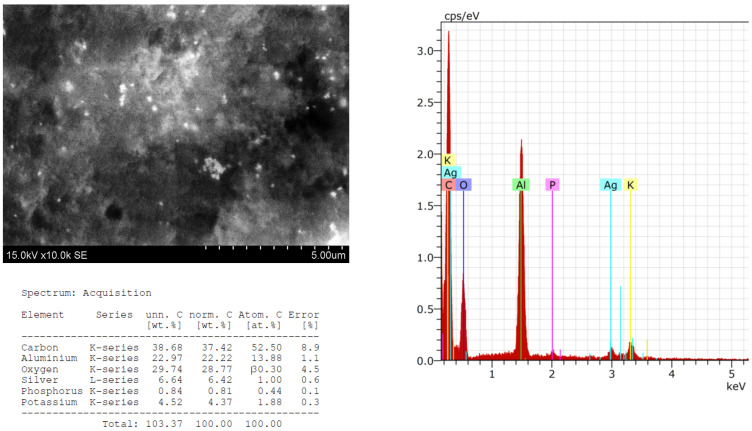
SEM images and EDS spectra of AgNPs synthesized using extract of *S. officinalis*.

The morphology, size, shape, and chemical composition of biosynthesized HysO-AgNPs and SalO-AgNPs were examined by scanning electron microscope and energy dispersive X-ray spectrometer (SEM-EDS) techniques. SEM images and EDS spectra of AgNPs, green-synthesized using extracts of HysO and SalO, are presented in Figure 3 and Figure 4, which confirm the formation of AgNPs. EDS spectra and elemental analysis are also presented, where potassium, chlorine, magnesium, and silver are also visible.

EDS spectra show peaks at 3.0 keV, which can be attributed to the binding energy of silver [36]. It can be seen that the AgNPs are evenly distributed throughout the studied area (Figure 3).

The formation of SalO-AgNPs is confirmed by SEM images and the EDS spectrum (Figure 4). Single, spherical nanoparticles are faintly visible. In this case, we can already see a tendency to agglomerate in places. This can be associated with the chosen form of analysis of metal particles when the particles were poured onto aluminum foil, dried, and then analyzed, which probably influenced the formation of agglomerates. However, it is still observed that the particles are distributed throughout the apparent area.

**Figure 5:**
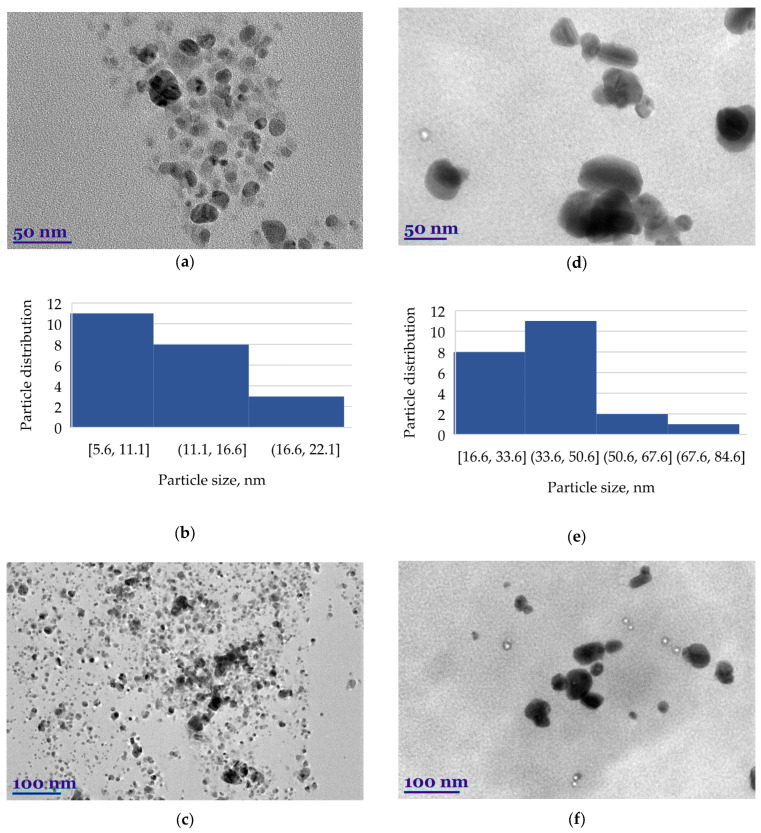
TEM images of biosynthesized by *H. officinalis* silver nanoparticles (HysO-AgNPs) (**a**,**c**); and biosynthesized by *S. officinalis* silver nanoparticles (SalO-AgNPs) (**d**,**f**); and particle size distribution histograms of HysO-AgNPs (**b**) and SalO-AgNPs (**e**).

Transmission electron microscope (TEM) spectra confirm the presence of biosynthesized AgNPs (Figure 5). The morphology, shape, size, and size distribution of green biosynthesized HysO-AgNPs and SalO-AgNPs are shown.

The analysis indicated that the particles were mainly spherical but also detected to be triangular or hexagonal; other irregular geometrically shaped particles were also visible. A slight agglomeration of AgNPs can be seen at 100 nm magnification, but at 50 nm magnification, the agglomerates are not identified (Figure 5). SalO-AgNPs are basically spherical in shape, but as we can see in the photo of Figure 5d, there are also cylindrical, irregular, and oval-shaped particles. ImageJ application was used to determine the size of the AgNPs. The size distribution of green-synthesized AgNPs is presented in histograms (Figure 5b,e). Overall, HysO-AgNPs length was greater than those of SalO-AgNPs. The average size of HysO-AgNPs particles was 11.79 nm. In the case of SalO-AgNPs, the size was more than three times bigger—38.03 nm. After evaluation of green AgNPs size distribution, it was seen that the size of the nanoparticles depends on the plant extract used for stabilization and bio-reduction.

### 2.4. Molecular Identification of Fusarium spp.

**Figure 6:**
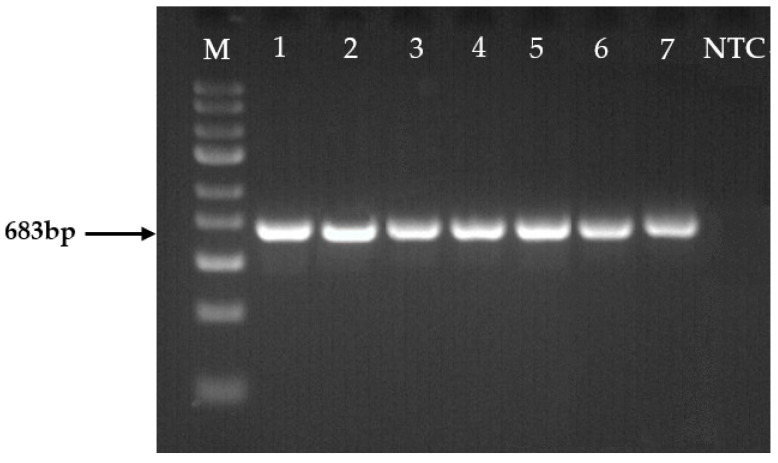
Isolates of *Fusarium* spp. amplified with the *TEF1α* gene. Lanes: M, molecular weight marker GeneRuler Express DNA Ladder (Thermo Fisher Scientific Baltics, Vilnius, Lithuania); 1, *Fp*; 2, *Fo1*; 3, *Fo2*; 4, *Fa2*; 5, *Fc*; 6, *Fa1*; 7, *Fg*; NTC, negative template control.

*Fusarium* spp. was isolated from six different host-plants: strawberry, carrot, asparagus, pea, rapeseed, and spring wheat (Table 2). Individual species were identified by amplifying each strain’s partial translation elongation factor 1-alpha (*TEF1α*) gene (Figure 6). The *TEF1α* partial gene was successfully amplified in all strains, yielding a single band in size of about 683 base pairs (bp).

The *TEF1α* gene sequences from each of the 7 *Fusarium* isolates were compared with those from various species of *Fusarium* in the NCBI database. The results revealed that of all strains, 28.6% belonged to *F. oxysporum*, 28.6% to *F. avenaceum*, and 14.3% each to *F. proliferatum*, *F. culmorum*, and *F. graminearum* (Table 2).

### 2.5. Inhibition of Fusarium spp. In Vitro

**Figure 7:**
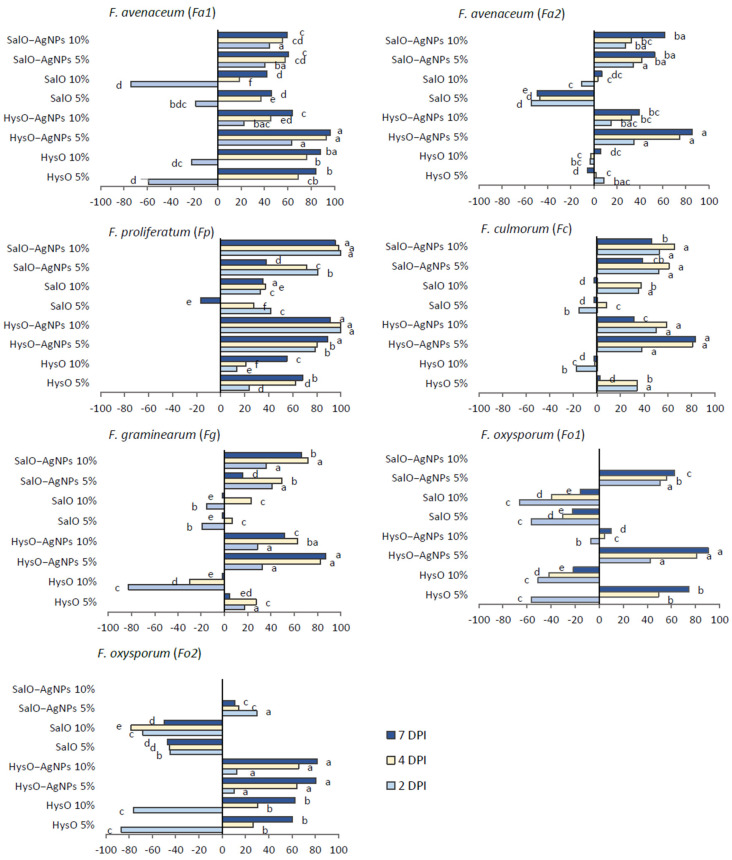
Inhibition (%) of *Fusarium* spp. after treatment with: *H. officinalis* extract 5% (HysO 5%); *H. officinalis* extract 10% (HysO 10%); *H. officinalis* extract based AgNPs 5% (HysO-AgNPs 5%); *H. officinalis* extract based AgNPs 10% (HysO-AgNPs 10%); *S. officinalis* extract 5% (SalO 5%); *S. officinalis* extract 10% (SalO 10%); *S. officinalis* extract based AgNPs 5% (SalO-AgNPs 5%); *S. officinalis* extract based AgNPs 10% (SalO-AgNPs 10%) at 2, 4 and 7 days post inoculation (DPI). 5% and 10% concentration specify added pure obtained solutions of aqueous extracts and AgNPs (*v*/*v*) in the medium. According to Duncan’s multiple range test, different letters indicate statistical differences between the means (*p* < 0.05).

The inhibition of *Fusarium* spp. under the treatment of hyssop and sage extracts and green-synthesyzed AgNPs is shown in Figure 7.

Among investigated *Fusarium* fungi, *Fa*1, *Fp,* and *Fo*2 were the most sensitive to both concentrations of HysO. SalO caused lower inhibition and was the most effective in inhibiting *Fa*1, *Fp*, and *Fc*. Investigated *Fusarium* spp. were inhibited the most by adding SalO-AgNPs 10% and HysO-AgNPs 5% solutions to the medium. Comparing all *Fusarium* fungi, *Fp* was the most sensitive to all treatments. Despite some variance between investigated extracts concentrations in the medium, SalO and HysO demonstrated a tendency to inhibit the growth of *Fusarium* spp. in vitro. Overall, AgNPs based on both extracts expressed the highest antifungal activity and showed the ability to control *Fusarium* spp. fungal growth.

## 3. Discussion

Modern agriculture requires sustainable methods in all production steps, including crop protection. Due to chemical fungicides being undesired, various other substances are under investigation. Silver nanoparticles (AgNPs) are well known for their antimicrobial properties, and with green synthesis, plant extracts could be used to produce them. This study investigated AgNPs synthesized using *S. officinalis* and *H. officinalis* extracts, and pure *S. officinalis* and *H. officinalis* extracts, used for AgNPs reduction. SalO and HysO previously demonstrated efficacy in controlling fungi growth in vitro [11,15,17,37]. Additionally, considering that in one of the studies, lower concentrations of these extracts were used [17], resulting in a moderate antifungal effect, in our case, the choice of higher concentrations increased the probability of significant results.

The biological activity of synthesized AgNPs is characterized by their phytochemical composition and antioxidant activity. Proanthocyanidins, hydroxycinnamic acid, phenolics, and flavonoids from plant extracts participate in the synthesis as natural reductors, anticoagulants, and stabilizers instead of chemical compounds being used. Other studies found increased flavonoids and total phenolic compounds after the green synthesis of AgNPs [38]. However, we found a reduction of these compounds in our case. As SalO and HysO resulted well in AgNPs formation, quantities of phytochemicals such as proanthocyanidins, hydroxycinnamic acid derivatives, phenolic compounds, and flavonoids decreased. This could be an explanation for the absence of an increase in antioxidant capacity as components of natural SalO and HysO, responsible for antioxidant properties, were also used in the biosynthesis process. In our case, AgNPs had almost the same antioxidant and ferric-reducing activity as the extracts used for synthesis. A slight reduction of ferric reducing antioxidant activity of SalO-AgNPs is similar to that described in calendula extract-mediated AgNPs [27]. Contrarily, green-synthesized AgNPs had much higher DPPH radical scavenging and FRAP antioxidant activities than crude fruit extract of *P. farcta* [36]. HysO extract based AgNPs had slightly higher activity against DPPH• and ABTS•+ compared to pure extracts, which is consistent with previous research [27]. Similar results were also obtained with sage extracts previously [26].

Widely found filamentous fungi of the large genus *Fusarium* are responsible for plant diseases and food contamination with mycotoxins [28]. In addition, numerous species are pathogenic and cause severe diseases in different plant hosts of agronomic importance. Our study identified seven fungal strains isolated from six host plants by amplifying and sequencing the *TEF1α* partial gene. Characteristic species such as *F. graminearum* (Fusarium head blight) to spring wheat [39], *F. oxysporum* to peas and rapeseeds (Fusarium wilt) [40], *F. avenaceum* to asparagus (Fusarium wilt) [28] were identified. Other less common fungi were isolated from other isolates. *Fusarium* communities can vary in terms of species diversity, depending on crops, soil physicochemical characteristics, climatic conditions, human activities, and other factors [41].

Antioxidant activity could be closely related to the antibacterial and other biological effects of plant extract and AgNPs. Despite medium antioxidant capacity, both our produced AgNPs expressed high antifungal activity on *Fusarium* spp. In this study, inhibition of *Fusarium* spp. radial colony growth was determined in vitro under treatments with different concentrations of prepared extracts and of the AgNPs produced using the *H. officinalis* and *S. officinalis* plant leaves’ extracts as eco-friendly reducing agents. Our results agree with the research, the AgNPs obtained with *Azadirachta indica* leaf extract also demonstrated antifungal activity against *Fusarium* spp. [42]. The mechanism of antibacterial activity of biosynthesized AgNPs is explained as possible shutdown of DNA replication and degradation of cell membranes [43]. Our results revealed that comparing two investigated AgNPs, HysO-AgNPs more efficiently inhibited *Fusarium* spp. According to Salem and Fouda [22], comparable to analogous nanostructures, the physicochemical properties of AgNPs depend on their size and shape, which rely on the chemical composition and functional groups that exist in the plant extract. SEM and TEM analysis showed the distribution of AgNPs, demonstrated the occurred synthesis, and clarified the size, aggregation, and morphological shapes of green synthesized AgNPs. Based on the results, HysO and SalO are eligible green reductors and could be used instead of chemical ones. In our study, after evaluating the particle distribution using the ImageJ application, it was found that the size of the AgNPs depends on the plant aqueous extract used for bio-reduction and stabilization. Banerjee and colleagues [44] suggest that the presence of different quantities and nature of capping agents in the extracts could influence the shape differences in AgNPs solutions. Overall, the size of HysO-AgNPs was greater than that of SalO-AgNPs, which may affect differences in antifungal activity. Biofabricated using plant extracts, AgNPs with an average size above 100 nm exhibited significant bacteria growth inhibition at 20% formulation [45]. In contrast, increased concentration, and decreased nanometer size of AgNPs positively impacted antifungal activity on *F. oxysporum* [4]. In addition, biosynthesized AgNPs reduced *F. graminearum* mycelium growth, spore germination rate, germ tube growth and caused the leakage of the pathogen’s DNA [31]. Our results agree with this study, as both hyssop and sage extract based AgNPs inhibited the growth of *F. graminearum* and were more effective than pure aqueous extracts.

Various plant extracts have recently shown fungal growth inhibition. However, AgNPs based on plant extracts may result in even higher fungal growth suppression and could have a high potential to be used for the development of antifungal substances. Overall, the biological activity of AgNPs based on plant extracts could be versatile and highly dependent on the green reductor. To conclude our results, AgNPs based on hyssop and sage extracts showed high potential to inhibit *Fusarium* fungi, especially isolates from agricultural crops. Moreover, our results suggest that plant species as a different biological compound matrix directly impact the nanoparticles’ morphology, size, size distribution, and related biological activities.

## 4. Materials and Methods

### 4.1. Preparation of the Extracts and Green Synthesis of AgNPs

Dried *Salvia officinalis* L. and *Hyssopus officinalis* L. leaves were purchased from the University pharmacy LSMU (Kaunas, Lithuania) and Svencioniu vaistazoles (Svencionys, Lithuania) and ground to powder. 20 g raw powder was poured with 200 mL distilled water and left to macerate for 24 h in the dark at room temperature. Aqueous extracts of SalO and HysO were collected and kept at −20 °C until further use. 0.03 g of AgNO_3_ (≥99%) was mixed while stirring with 30 mL of each prepared aqueous extract. Mixtures were left for 24 h in the dark, and 42 °C while stirring 80 min. to form AgNPs, and collected extracts were refrigerated until further experiments.

### 4.2. Phytochemical Composition of Prepared Extracts

Phytochemical composition was determined in triplicates for all samples. The total amount of proanthocyanidins was determined using the 4-dimethylaminocinnamaldehyde (DMAC) method with Shimadzu UV-1800 spectrophotometer at 640 nm as stated in [46]. The results were expressed as epicatechin equivalents per dry weight (DW) (mg EE/100 g DW). The total amount of hydroxycinnamic acid derivatives was analyzed according to [47] by reading absorption at 525 nm and expressed as mg chlorogenic acid equivalent (CAE) per one gram of dry weight (mg CAE/g DW). The total amount of phenolic compounds was analyzed according to the Folin-Ciocalteu method, using gallic acid as the standard [46]. Absorbance was measured at 765 nm using UV-Vis spectrophotometer Genesys 10 UV. Results expressed as mg/100 gallic acid equivalent (GAE) per gram of dry weight (mg GAE/g DW). The total amount of flavonoids was determined by by measuring absorbance after 25 min incubation at 430 nm [48] and expressed as mg rutin equivalent (RE) per gram of dry weight (mg RE/g DW).

### 4.3. Antioxidant Capacity

Different antioxidant activity assays were performed to describe the antioxidant capacity of investigated extracts and produced AgNPs. ABTS•+ radical cation decolorization was performed as described [49]. DPPH• free radical scavenging activity was analyzed according to Puzeryte et al. [46]. Absorbance of samples were measured at 734 nm for ABTS and 515 nm for DPPH using UV-Vis spectrophotometer Genesys 10 UV. Ferric reducing antioxidant power (FRAP) was analyzed by the method according to Raudone et al. [50]. Reagents mixture with 20 µL of sample was incubated for 60 min and absorbance measured at 593 nm. Antioxidant activity was calculated from Trollox (Merck KGaA, Darmstadt, Germany) calibration curve and expressed as mg TE mL^−1^ of extract.

### 4.4. Structural and Qualitative Analysis of Silver Nanoparticles

The formation of HysO-AgNPs and SalO-AgNPs was studied employing a Lambda 25 UV-vis spectrometer (PerkinElmer, Waltham, MA, USA). The research was conducted in the wavelength spectrum from 200 to 800 nm. Ultrapure water was used as the blank for UV-Vis experiments.

Images of obtained green-synthesized AgNPs for morphological structure determination were obtained using scanning electron microscope (SEM) FEI Quanta 200 FEG (FEI Company, Hillsboro, OR, USA). A low-vacuum mode, 20.0 kV, and LDF detector were used to examine samples from three different locations at a resolution of 1.2 nm.

Transmission electron microscope (TEM) A Tecnai G2 F20 X-TWIN (FEI, Lausanne, Switzerland) was used to analyze the size and shape of AgNPs. Samples were diluted and deposited on TEM grids for analysis. The field emission electron gun had an accelerating voltage of 200 kV. Particle size was measured with the software ImageJ-win32 1.53k. Elemental analysis was performed using an energy-dispersive X-ray spectrometer (EDS) as described by Balciunaitiene et al. [27].

### 4.5. Isolation of Fusarium spp.

*Fusarium* spp. fungi were isolated from strawberry (*Fragaria × ananassa* Duchesne), carrot (*Daucus sativus* (Hoffm.) Röhl.), asparagus (*Asparagus officinalis* L.), rapeseed (*Brassica napus* L.), pea (*Pisum sativum* L.) and spring wheat (*Triticum aestivum* L.) host-plants that were collected from different Lithuanian regions. Isolates were grown on a potato dextrose agar (PDA) medium at 25 ± 2 °C for 7–10 days in the dark. Then transferred on Spezieller Nährstoffarmer Agar (SNA) [51] and incubated under the same conditions until the formation of macroconidia. Spore suspensions of each isolate were spread onto 2% water agar, from which single-spore isolates were picked and subcultured on PDA and SNA. Morphological characteristics were studied using light microscopy [51].

### 4.6. DNA Amplification and Sequencing

Seven potential isolated strains of *Fusarium* spp. were selected for molecular identification. The *TEF1α* gene was amplified directly from fungal samples by using primers EF1 (5’-ATGGGTAAGGARGACAAGAC-3’) and EF2.1 (5’-GGARGTACCAGTSATCATGTT-3’) [52], and a PhireTM Plant Direct PCR Master Mix (Thermo Fisher Scientific, Waltham, MA, USA) according to the manufacturer’s instructions. Amplifications were performed in a 20 μL total volume using a thermal cycler (Eppendorf, Hamburg, Germany) with initial denaturation of 5 min at 98 °C, followed by 35 cycles of 5 s denaturation at 98 °C, 5 s annealing at 58.3 °C. The thermal cycles were terminated by an extension for 20 s at 72 °C and a final extension for 1 min at 72 °C. 6 μL of PCR-amplified products were visualized on 1.5% agarose gels in 1 × TAE buffer, stained with Midori Green Advance (Nippon Genetics Europe, Düren, Germany) at 80 V cm^−1^.

DNA was purified using an Exo-CIP Rapid PCR Cleanup Kit (New England Biolabs GmbH, Frankfurt am Main, Germany) according to the manufacturer’s instructions and prepared for sequencing by mixing 10 µL of cleaned product, 2 μL of PCR water and 2 μL of EF22T (5’-AGGAACCCTTACCGAGCTC-3’) primer. The amplified DNA fragments were sequenced by LGC Genomics GmbH (Berlin, Germany) and analyzed using the BLAST tool 2.13.0 from the National Center for Biotechnology Information (https://blast.ncbi.nlm.nih.gov/Blast.cgi, accessed on 22 May 2023) to confirm their identities [53].

### 4.7. In Vitro Experiments

The potato dextrose agar medium was prepared under recommendations and sterilized. Cooled medium (around 45 °C) was separately mixed with calculated volumes of sage and hyssop aqueous extracts and sage and hyssop based AgNPs to make 5 and 10% concentrations (*v*/*v*) in the medium. Medium mixtures were poured into Petri dishes. 7 mm diameter fungal discs were cut from investigated *Fusarium* colonies and placed in the center of Petri dishes. No extract was added in control treatments. Incubation was performed at 22 °C in the dark. Radial colony growth of each fungus was measured in cm and used for calculation of the inhibition [54]: Mycelial growth inhibition (%) = (*C* − *T*)/*C*, where *C* is radial growth of the pathogen colony in control (cm), *T*—radial growth of the pathogen colony in the treatment (cm).

### 4.8. Statistical Analysis

Results were analyzed using the SAS Enterprise Guide 7.1 program (SAS Institute, Cary, NC, USA). Duncan’s multiple range test (*p* < 0.05) was performed to determine the significant differences between the means of treatments.

## Figures and Tables

**Table 1:** Phytochemical analysis of *H. officinalis* (HysO) and *S. officinalis* (SalO) extracts and silver nanoparticles synthesized by *H. officinalis* extract (HysO-AgNPs) and *S. officinalis* extract (SalO-AgNPs).

Compounds	HysO	SalO	HysO-AgNPs	SalO-AgNPs
The total amount of proanthocyanidins, mg EE/g DW	0.15 ± 0.03	0.18 ± 0.01	0.08 ± 0.01	0.12 ± 0.01
The total amount of hydroxycinnamic acid derivatives, mg CAE/g DW	1.09 ± 0.02	1.51 ± 0.04	0.86 ± 0.03	1.42 ± 0.03
The total amount of phenolic compounds, mg GAE/g DW	1.17 ± 0.00	0.92 ± 0.01	0.79 ± 0.00	0.73 ± 0.01
The total amount of flavonoids, mg RE/g DW	0.58 ± 0.01	0.31 ± 0.01	0.35 ± 0.02	0.27 ± 0.01

Data is presented as mean ± standard deviation. EE—epicatechin equivalents; DW—dry weight; CAE—chlorogenic acid equivalent; GAE—gallic acid equivalent; RE—rutin equivalent.

**Table 2:** Identification of *Fusarium* spp. associated with host-plants based on *TEF1α* gene sequencing.

S. No.	Host Plant	Isolate Code	Identification by *TEF1α* Gene	NCBI Accession No.
1.	Strawberry *(Fragaria × ananassa* Duch.)	*Fp*	*Fusarium proliferatum*	JX118976.1
2.	Pea (*Pisum sativum* L.)	*Fo*1	*Fusarium oxysporum*	MG356947.1
3.	Rapeseed (*Brassica napus* L.)	*Fo*2	*Fusarium oxysporum*	MG857278.1
4.	Asparagus (*Asparagus officinalis* L.)	*Fa*2	*Fusarium avenaceum*	MG857265.1
5.	Carrot (*Daucus sativus* (Hoffm.) Röhl.)	*Fc*	*Fusarium culmorum*	MG857178.1
6.	Strawberry (*Fragaria × ananassa* Duch.)	*Fa*1	*Fusarium avenaceum*	MG857265.1
7.	Spring wheat (*Triticum aestivum* L.)	*Fg*	*Fusarium graminearum*	MG857515.1

## Data Availability

Data is contained within the article.
